# Recombinant Modified Vaccinia Virus Ankara (MVA) Vaccines Efficiently Protect Cockatiels Against Parrot Bornavirus Infection and Proventricular Dilatation Disease

**DOI:** 10.3390/v11121130

**Published:** 2019-12-06

**Authors:** Isabell Rall, Ralf Amann, Sara Malberg, Christiane Herden, Dennis Rubbenstroth

**Affiliations:** 1 Institute of Virology, Medical Center–University of Freiburg, Faculty of Medicine, University of Freiburg, Hermann-Herder-Str. 11, D-79104 Freiburg, Germany; isabell.rall@web.de; 2 Department of Immunology, Interfaculty Institute of Cell Biology, Eberhard Karls Universität Tübingen, Auf der Morgenstelle 15, D-72076 Tübingen, Germany; Ralf.Amann@ifiz.uni-tuebingen.de; 3 Institute for Veterinary Pathology, University Justus Liebig Gießen, Frankfurter Str. 96, D-35392 Gießen, Germany; Sara.Malberg@vetmed.uni-giessen.de (S.M.); Christiane.Herden@vetmed.uni-giessen.de (C.H.); 4 Institute of Diagnostic Virology, Friedrich-Loeffler-Institut, Südufer 10, D-17493 Greifswald–Insel Riems, Germany

**Keywords:** avian bornaviruses, parrot bornavirus 2 (PaBV-2), parrot bornavirus 4 (PaBV-4), recombinant viral vector vaccines, modified vaccinia virus Ankara (MVA), proventricular dilatation disease (PDD)

## Abstract

Parrot bornaviruses (PaBVs) are the causative agents of proventricular dilatation disease (PDD), a chronic and often fatal neurologic disorder in Psittaciformes. The disease is widely distributed in private parrot collections and threatens breeding populations of endangered species. Thus, immunoprophylaxis strategies are urgently needed. In previous studies we demonstrated a prime-boost vaccination regime using modified vaccinia virus Ankara (MVA) and Newcastle disease virus (NDV) constructs expressing the nucleoprotein and phosphoprotein of PaBV-4 (MVA/PaBV-4 and NDV/PaBV-4, respectively) to protect cockatiels (*Nymphicus hollandicus*) against experimental challenge infection. Here we investigated the protective effect provided by repeated immunization with either MVA/PaBV-4, NDV/PaBV-4 or Orf virus constructs (ORFV/PaBV-4) individually. While MVA/PaBV-4-vaccinated cockatiels were completely protected against subsequent PaBV-2 challenge infection and PDD-associated lesions, the course of the challenge infection in NDV/PaBV-4- or ORFV/PaBV-4-vaccinated birds did not differ from the unvaccinated control group. We further investigated the effect of vaccination on persistently PaBV-4-infected cockatiels. Remarkably, subsequent immunization with MVA/PaBV-4 and NDV/PaBV-4 neither induced obvious immunopathogenesis exacerbating the disease nor reduced viral loads in the infected birds. In summary, we demonstrated that vaccination with MVA/PaBV-4 alone is sufficient to efficiently prevent PaBV-2 challenge infection in cockatiels, providing a suitable vaccine candidate against avian bornavirus infection and bornavirus-induced PDD.

## 1. Introduction

Avian bornaviruses belong to the family *Bornaviridae* and are widely distributed in captive populations of parrots and related species (Psittaciformes) [1,2]. To date, eight parrot bornaviruses (PaBVs) have been discovered in psittacine birds, belonging to the viral species *Psittaciform 1 orthobornavirus* (encompassing PaBV-1 to PaBV-4, PaBV-7 and tentatively PaBV-8) and *Psittaciform 2 orthobornavirus* (PaBV-5 and tentatively PaBV-6) [3,4]. PaBVs establish persistent infections in their hosts with highly variable clinical outcomes. In some individuals, a PaBV infection causes proventricular dilatation disease (PDD), a peracute to chronic, often fatal disease usually associated with neurological and/or intestinal symptoms. In contrast, other persistently infected psittacines show no clinical symptoms for up to several years [5,6,7,8,9,10]. Immunopathogenesis is assumed to be required for the development of PDD, providing a possible explanation for this pattern [6]. In line with this assumption, PDD in experimentally infected cockatiels was prevented by immunosuppressive treatment with cyclosporine A (CsA) at the time of PaBV-2 inoculation [11]. This is in congruence with the closely related mammalian Borna disease virus 1 (BoDV-1; species *Mammalian 1 orthobornavirus*), which causes T cell-mediated immunopathogenesis in various mammalian hosts [12,13,14,15].

At present, neither effective therapies nor immunoprophylaxis are available for avian bornavirus infection and PDD despite their considerable impact on private psittacine collections as well as on breeding projects of endangered species [16,17]. In order to protect psittacines against avian bornavirus infections, we previously generated recombinant modified vaccinia virus Ankara (MVA; family *Poxviridae*) and Newcastle disease virus (NDV; family *Paramyxoviridae*) vaccines expressing the nucleoprotein (N) and phosphoprotein (P) of PaBV-4 [6]. A combination of both vaccines protected cockatiels (*Nymphicus hollandicus*) against challenge infection with the closely related PaBV-2 and against PDD-associated lesions (Runge et al., 2017). However, the individual contribution of each viral vector had not been determined. In this study, we evaluated the protective effect provided by vaccination of cockatiels with either MVA or NDV constructs alone. In addition, a newly generated set of Orf virus (ORFV; family *Poxviridae*) vector vaccines [18] expressing PaBV-4 N and P was included. ORFV-based recombinant vector vaccines have been successfully applied in a broad range of species [19,20,21], including efficient protection of rats against experimental BoDV-1 infection [13,22].

In the second part of the study, the effect of vaccination on an established persistent infection was evaluated. Therefore, cockatiels experimentally infected with PaBV-4 were subsequently vaccinated with MVA and NDV constructs to investigate whether vaccination induces immunopathogenesis or contributes to reduction of viral loads.

## 2. Materials and Methods

### 2.1. Viruses

PaBV-4 #6758 (GenBank accession number JX065209) and PaBV-2 #17684 (JX065197) were isolated from a blue-and-gold macaw (*Ara ararauna*) or a cockatiel (*Nymphicus hollandicus*), respectively, suffering from PDD [23]. NDV and MVA vaccine constructs expressing the N or P genes of PaBV-4 #6758 (rNDV/PaBV-4/N, rNDV/PaBV-4/P, rMVA/PaBV-4/N and rMVA/PaBV-4/P) have been described in detail elsewhere [6]. The parental strains MVA-F6 [24] and recombinant NDV clone 30 [25] were kindly provided by Gerd Sutter, Munich and Angela Römer-Oberdörfer, Greifswald-Riems, respectively. Following previously published procedures, bornavirus stocks were prepared from persistently infected QM7 quail muscle or CEC-32 quail fibroblast cultures [9,26]. MVA stocks were produced in primary chicken embryo fibroblasts and NDV viruses in embryonated chicken eggs [6].

### 2.2. Generation of ORFV Constructs Encoding Avian Bornavirus N and P Genes

Two recombinant ORFV vaccine constructs carrying either the N or P gene of PaBV-4 #6758 (designated rORFV/PaBV-4/N and rORFV/PaBV-4/P, respectively) were generated based on the attenuated vector D1701-V-CD4-D12-mCherry as previously described [18,27,28]. Briefly, the open reading frames (ORF) of the bornavirus genes were inserted into transfer plasmids (Figure S1). Subsequently, Vero African green monkey kidney cells, infected with the parental ORFV virus, were transfected with the transfer plasmids. Negative magnet-associated cell sorting and limiting dilution series were used to select for recombinant viruses, in which the bornavirus ORF had replaced the CD4 marker gene by homologous recombination [18,28]. Stocks of purified rORFV viruses were generated in Vero cells by three freeze-thawing cycles and ultracentrifugation [18]. The correct gene insertion was confirmed by PCR of selected genome regions. The bornavirus antigens were expressed under the control of an ORFV-specific early promotor. Hence, viral replication is not required for antigen expression. The expression of bornavirus N or P proteins and the ability of the ORFV constructs to infect avian cells was demonstrated by immunofluorescence staining, flow cytometry and Western blotting (Figures S2 and S3).

### 2.3. Immunization and Experimental Bornavirus Infection of Cockatiels

In a first experiment, 16 cockatiels aged 10 to 16 months were divided into four groups of four cockatiels each. One group (NDV) was vaccinated with equal amounts of rNDV/PaBV-4/N and rNDV/PaBV-4/P (10^6.9^ focus-forming units [ffu] of each virus per bird), a second group (MVA) was vaccinated with a mixture of rMVA/PaBV-4/N and rMVA/PaBV-4/P (10^7.9^ ffu), and a third group (ORFV) received a mixture of rORFV/PaBV-4/N and rORFV/PaBV-4/P (10^7.0^ ffu). Two and four weeks later, all three groups received two booster vaccinations with the same doses of the corresponding vaccines. All vaccinations were performed by intramuscular injection of 200 µl vaccine preparation per bird. Three weeks after the last booster vaccination, a fourth group of four non-vaccinated birds (Control) was added and all groups received a challenge infection with 10^3.5^ ffu of PaBV-2 #17684 per bird by combined intramuscular and subcutaneous injection of 200 µl virus preparation per bird. Until the challenge infection, the NDV-vaccinated birds were housed in a separate aviary, while MVA- and ORFV-vaccinated birds were housed together. After challenge, all groups shared the same aviary. The experiment was terminated 16 weeks after challenge infection.

In a second experiment, 12 cockatiels aged eight to 20 months were inoculated with 10^5.1^ ffu of PaBV-4 #6758 per bird by combined intramuscular and subcutaneous injection. Twelve weeks after infection, the birds where divided into two separately housed groups of six birds each. Both groups were designed to be comparable regarding shedding of viral RNA, seroconversion, bodyweight, sex, age and signs of clinical disease (Figure S6). Subsequently, the birds received three intramuscular injections of 200 µl vaccine preparation each. The immunized group was vaccinated with rMVA/PaBV-4/N and P (10^8.1^ ffu of each construct per bird and vaccination) during weeks 12 and 18 and with rNDV/PaBV-4/N and P (10^7.1^ ffu) in week 15. In parallel, the control group received similar doses of the respective parental vaccine strains not expressing bornavirus antigens. The experiment was terminated 22 weeks after PaBV-4 inoculation, equaling ten weeks after the first vaccination.

Clinical signs were recorded daily throughout both experiments. At intervals of one to two weeks, bodyweights were determined and cloacal swabs were collected for detection of bornavirus- and vaccine-derived nucleic acids. Serum samples were collected for detection of bornavirus-reactive antibodies as well as antibodies directed against the MVA and NDV vectors. Animals were euthanized when sustained weight loss or severe disease was observed. All remaining birds were euthanized at the end of the experiments and organ samples were collected for virus detection and histopathological analysis.

All animal experiments were performed in compliance with the German animal protection law (TierSchG) and were approved by the ethical committee of the local authorities (Regierungspräsidium Freiburg; application number 35-9185.81/G-13/55, permission date 7 June 2013). The animals were housed and handled in accordance with good animal practice as defined by FELASA (http://www.felasa.eu) and the national animal welfare body GV-SOLAS (http://www.gv-solas.de). Housing conditions have been described in detail elsewhere [6,9].

### 2.4. Detection of PaBV-2- and PaBV-4-Specific RNA by RT-qPCR Assays

Viral RNA was extracted from cloacal swabs and organ samples using QIAamp viral RNA mini kit (Qiagen, Hilden Germany) or Trifast (Peqlab, Erlangen, Germany), respectively. Following reverse transcription (RT) by Revertaid, reverse transcription reagents (Thermo Scientific, Schwerte, Germany), bornavirus-specific RNA was quantified by two TaqMan quantitative PCR (RT-qPCR) assays specific for either PaBV-4 [29] or PaBV-2 [10]. Threshold cycle (ct) values below 37 were considered positive. The cDNA equivalent of 0.1 µg tissue RNA or 0.5% of the total RNA from swab material was included per qPCR reaction. Viral RNA copy numbers were calculated using standard dilution series of plasmids containing the complete PaBV-2 or PaBV-4 P gene. All procedures have been described in detail elsewhere [9,10,26].

### 2.5. Detection of Antibodies Directed Against Bornaviruses, NDV and MVA

Bornavirus-reactive antibodies were quantified by the indirect immunofluorescence test (iIFT) using QM7 cells persistently infected with PaBV-4 #6758 as target cells. Results were calculated as endpoint titres per ml serum. NDV-specific antibodies were determined by hemagglutination inhibition (HAI) assay and are presented as log_2_ HAI units. Detailed procedures for both tests have been described elsewhere [6,9,30].

Detection of antibodies directed against MVA by iIFT was performed as follows: Confluent QM7 cells layers in 96-well plates were inoculated with MVA-F6 at a multiplicity of infection of 0.25. Wells receiving virus-free infection medium served as negative controls. After incubation for 20 h, the cells were fixed with 4% paraformaldehyde and subsequently permeabilized with 0.5% Triton X-100. Two-fold dilution series of serum samples were prepared in Tris-buffered saline with Tween 20, pH 8.0 (T9039, Sigma-Aldrich, Taufkirchen, Germany) and 50 µl of each dilution were added in parallel to MVA-positive and -negative wells. After incubation for 60 min, the plates were washed three times with phosphate-buffered saline (PBS) and incubated for another 60 min with rabbit-anti-grey parrot IgG (kindly provided by Prof Bernd Kaspers, Munich, Germany) diluted in T9039 buffer, followed by an additional washing cycle with PBS and incubation for another 60 min with goat-anti-rabbit IgG conjugated with Cy3 (Jackson Immunoresearch). After a final washing cycle, the wells were analysed by fluorescence microscopy and for each serum dilution, MVA-positive and -negative wells were compared. Wells were considered positive if the expected approximately 30% MVA-positive cells were clearly distinguishable from the background staining of uninfected cells in the same well and in the corresponding MVA-negative control well. All tests were performed with a minimal dilution factor of 10. Samples without detection of a specific signal were assigned a titre of <10.

### 2.6. Histopathological Analysis

Tissue samples were stained with haematoxylin and eosin and subsequently analysed by two experienced pathologists (C.H., S.M.), who were blinded to the identity of the samples. Scores were assigned as “-” (no or only physiological levels of mononuclear cells present in tissues and ganglia), “+”, “++” or “+++” (mild, moderate or severe mononuclear infiltration and ganglioneuritis) in a semi-quantitative analysis.

## 3. Results

### 3.1. Expression of N and P Protein in Mammalian and Avian Cells Infected with Recombinant ORFV Vector Vaccines

Newly generated recombinant ORFV constructs expressing the N and P genes of PaBV-4 (designated rORFV/PaBV-4/N and rORFV/PaBV-4/P) were confirmed to express the desired bornavirus antigens by immunofluorescence staining and Western blot (Figure S2). The ability of the ORFV constructs to infect avian cells was confirmed in cell culture. Although infection efficiency was lower for avian cells as compared to mammalian Vero cells (Figure S3A), all ORFV constructs readily infected chicken and quail cell lines and expressed the respective bornavirus antigens (Figure S3B,C).

### 3.2. MVA Vector Vaccines Carrying PaBV-4 N and P Genes Protect Cockatiels Against Heterologous PaBV-2 Challenge Infection

To evaluate the protective effect provided by recombinant NDV, MVA and ORFV vaccines expressing PaBV-4 antigens, three groups of four cockatiels were intramuscularly vaccinated three times in 2-week intervals with either NDV, MVA or ORFV constructs encoding PaBV-4 N and P. A fourth group served as an unvaccinated control. Three weeks after the last booster vaccination all groups received a heterologous PaBV-2 challenge infection.

**Figure 1:**
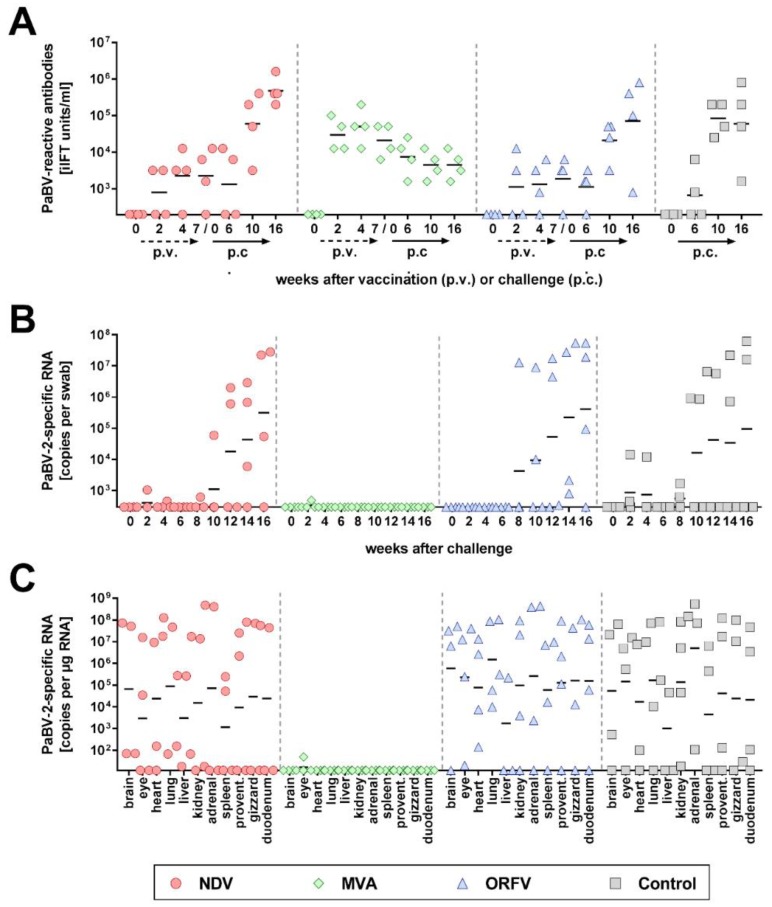
Modified vaccinia virus Ankara (MVA) vector vaccines expressing parrot bornavirus 4 (PaBV-4) nucleoprotein (N) and phosphoprotein (P) genes prevent persistent PaBV-2 challenge infection (experiment 1). Three groups (NDV, MVA, and ORFV) of four cockatiels each were immunized three times with the indicated vector vaccines expressing PaBV-4 N and P genes. A fourth group (Control) was not vaccinated. Three weeks after the third vaccination, all groups received a heterologous PaBV-2 #17684 challenge infection. (**A**) Serum samples were collected at the indicated time points after vaccination and after challenge infection. They were tested for PaBV-reactive antibodies by the indirect immunofluorescence test (iIFT). (**B**) Cloacal swabs were collected in two-week intervals after challenge infection and tested for PaBV-2-specific RNA by RT-qPCR. (**C**) All cockatiels were euthanized at 16 weeks after challenge infection and organ samples were analysed for PaBV-2-specific RNA by RT-qPCR. Results of individual birds are represented by dots. Horizontal lines indicate geometric means of the respective group. The position of the X axis indicates the detection limit of the respective test.

In accordance with previous findings [6], only a few birds showed transient shedding of low levels of vaccine-derived RNA or DNA following vaccination (Figure S4A,B). NDV- and MVA-vaccinated birds developed detectable levels of NDV-specific HAI antibodies or MVA-reactive iIFT antibodies, respectively, which were undetectable in the remaining groups (Figure S4C,D). Development of vaccine-induced PaBV-reactive antibodies was most prominent in the MVA-vaccinated birds, whereas the antibody levels induced by NDV and ORFV vaccines were considerably lower (Figure 1A).

During the first four weeks after challenge infection, two birds of the control group and one bird each of the NDV- or MVA-vaccinated group exhibited transient shedding of low levels of PaBV-2-specific RNA, which ceased until six weeks after challenge (Figure 1B). From week 8 after challenge infection onwards, three NDV-vaccinated birds, all four ORFV-vaccinated birds and two unvaccinated controls started shedding viral RNA, while none of the MVA-vaccinated cockatiels tested positive during this period (Figure 1B; Table 1). In congruence with their shedding profiles and similar to the unvaccinated control group, all birds of the NDV- and ORFV-vaccinated groups had viral RNA detectable in at least three organs collected at the end of the experiments. High copy numbers were detected in the organs of birds shedding high amounts of viral RNA, while birds without detectable virus shedding had only low to moderate copy numbers detectable in their organs (Figure 1C; Table 1). In contrast, only one bird of the MVA-vaccinated group had very low levels of PaBV-2-specific RNA detectable in the eye, whereas all other organs collected from this group tested negative (Figure 1C; Table 1). The course of seroconversion further confirmed the infection status of the birds. While the antibody titers of the NDV-vaccinated, ORFV-vaccinated and unvaccinated groups clearly increased after challenge infection, they gradually decreased in all birds of the MVA-vaccinated group, presumably due to the absence of persistent PaBV-2 infection in these birds (Figure 1A).

In agreement with the protection against challenge infection, the MVA-vaccinated birds remained almost completely free of inflammatory lesions in their tissues. In contrast, the majority of birds in the remaining groups developed non-suppurative ganglioneuritis or encephalitis (Table 1). One bird of the ORFV group (bird C2) exhibited signs of PDD, as characterized by marked weight loss and slight apathy beginning at fifteen weeks after challenge and severe proventricular dilatation observed at necropsy. No other bird showed apparent symptoms or gross lesions during this experiment (Table 1; Figure S5).

### 3.3. Immunization of Persistently PaBV-4-Infected Cockatiels Does Not Affect Course of Infection and Disease

**Figure 2:**
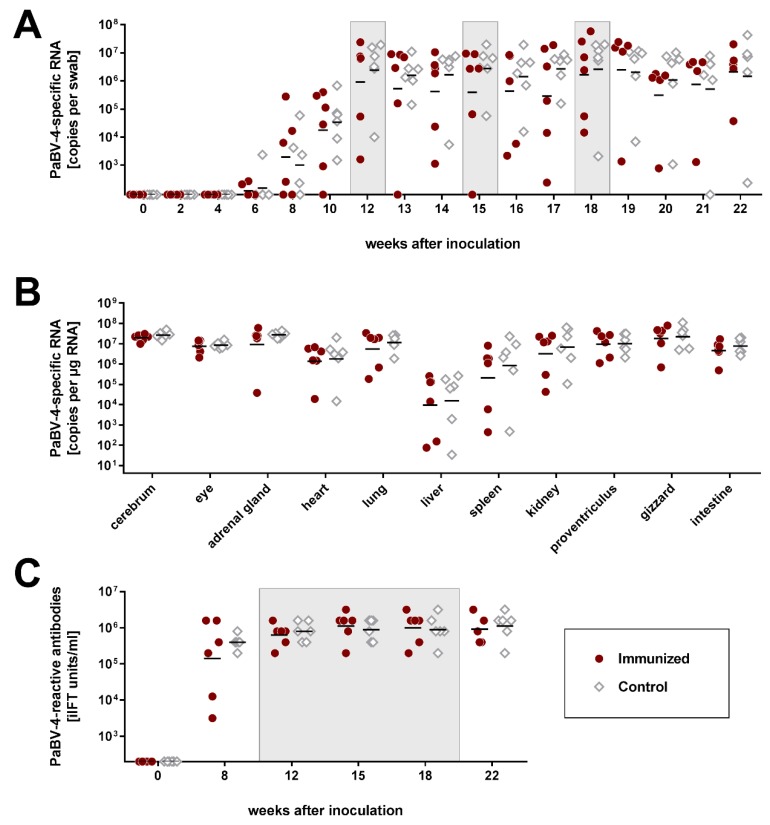
Immunization of persistently parrot bornavirus 4 (PaBV-4) -infected cockatiels does not affect the course of infection (experiment 2). Two groups of six cockatiels each were experimentally inoculated with PaBV-4 #6758. Subsequently, one group (Immunized) was immunized twice with mixtures of rMVA/PaBV-4/N and P at 12 and 18 weeks after infection and with rNDV/PaBV-4/N and P at 15 weeks after infection. The second group (Control) received the respective parenteral vaccine strains not expressing bornavirus antigens. Grey boxes indicate the time points of immunization. (**A**) Cloacal swabs were collected in two-week intervals after infection and weekly after immunization and tested for PaBV-4-specific RNA by RT-qPCR. (**B**) All cockatiels were euthanized at 22 weeks after PaBV-4 infection and organ samples were analysed for PaBV-4-specific RNA by RT-qPCR. (**C**) Serum samples were collected at the indicated time points and tested for PaBV-reactive antibodies by the indirect immunofluorescence test (iIFT). Results of individual birds are represented as dots. Horizontal lines indicate geometric means of the respective group. The position of the X axis indicates the detection limit of the respective test.

Studies on BoDV-1 infection in rodents suggest that immunization in the presence of an established bornavirus infection induces immunopathology and exacerbates disease [12,31]. On the other hand, vaccines may also help to reduce or even eliminate persisting viruses [32]. Thus, we next set out to investigate the efficacy and effect of our vaccines on persistently bornavirus-infected birds. Twelve subadult cockatiels were experimentally infected with a high dose of PaBV-4 #6758 (10^5.1^ ffu per bird) and divided into two equal groups twelve weeks later (Figure S6). At this time point, all birds had seroconverted and PaBV-4-specific RNA was detectable in their cloacal swabs (Figure 2A,B). Furthermore, five birds had transiently shown mild to moderate clinical signs until week 12 (Table 2; Figure S7A).

One of the groups (Immunized) was immunized three times with mixtures of rMVA/PaBV-4/N and P, rNDV/PaBV-4/N and P, and rMVA/PaBV-4/N and P again at 12, 15 and 18 weeks after infection, respectively, while the other group (Control) received the corresponding parental vaccines not expressing bornavirus antigens. The immune response to the vaccines was monitored by measuring the antibody response to the viral vectors. The observed NDV- and MVA-reactive antibody levels (Figure S8A,B) were slightly lower as compared to those achieved in the first experiment of this study (Figure S4C,D) but comparable to previous studies in which a vaccine-induced protective effect against parrot bornaviruses was achieved (Figure S8C,D) [6,10].

Despite induction of an immune response, the infection status of the birds was not affected. Amounts of PaBV-4 RNA detected in cloacal swabs or organ samples did not differ between the two groups (Figure 2A,B). One bird from each group (birds #5 and #7) that had already shown moderate signs of disease prior to immunization developed a more prominent course of disease thereafter (Figure S7A). Bird #5 of the immunized group had transiently shown mild to moderate apathy from week 6 to 8 after PaBV-4 infection, but recovered again and did not exhibit clinical signs after the first immunization with the rMVA/PaBV-4 constructs in week 12. However, in week 16, following the rNDV/PaBV-4 vaccination, the bird showed marked weight loss of 12.5%, followed by mild apathy and sudden death in week 18. Cockatiel #7 of the control group had shed undigested seeds prior to receiving the parental MVA vaccine in week 12. Thereafter, the bird continuously exhibited moderated apathy but was otherwise stable until the end of the experiment. One additional bird of each group (#6 and #10) transiently exhibited mild clinical signs starting two to three weeks after immunization with rNDV/PaBV-4 or the parental NDV vaccine, respectively (Figure S7A). Mononuclear infiltrations were detectable in tissues of all infected birds without apparent differences between the groups, but proventricular dilatation was not observed in any of the birds (Table 2; Figure S7B), indicating that the vaccination did not induce apparent immunopathology.

## 4. Discussion

We previously showed that infection-competent viral vector vaccines carrying PaBV-4 bornavirus N and P protect psittacine and passerine birds against avian bornavirus infections and subsequent disease. In these previous studies, MVA/PaBV-4 and NDV/PaBV-4 vaccine constructs were combined for heterologous prime-boost vaccination, but the individual protective potential of each viral vector was not scrutinized [6,10]. Here we demonstrate that application of MVA vaccines expressing both PaBV-4 N and P is sufficient to provide full protection against a PaBV-2 challenge infection of cockatiels, as determined by the absence of detectable viral RNA in cloacal swabs and tissues as well as by the absence of a detectable boost of bornavirus-reactive antibody titres after challenge. In congruence with immunity against PaBV-2 infection, the MVA/PaBV-4-vaccinated birds neither developed clinical PDD nor microscopic lesions suggestive of subclinical bornavirus-induced disease. In contrast, the corresponding NDV/PaBV-4 vaccines and a set of newly generated recombinant ORFV/PaBV-4 constructs failed to achieve protection under our experimental conditions. In agreement with its protective effect, MVA/PaBV-4 constructs induced the most prominent bornavirus-reactive humoral immunity among the tested vaccines. Detection of bornavirus-reactive antibodies served as a surrogate marker for adaptive immunity because tools for the detection of specific T lymphocytes are not available for cockatiels.

To achieve the most prominent effect, all vaccines in this study were used at the highest possible dose, as determined by the titres achieved for the vaccine preparations and the maximal injection volume per bird. Although viral titres of different viruses, which have been determined using different virus-dependent target cells and titration procedures, do not allow direct dose comparisons, the superior performance of the MVA constructs may be explained at least in part by the approximately ten-fold higher calculated dose titre as compared to NDV and ORFV. In addition, MVA was passaged more than 570 times in chicken embryo fibroblasts [24]. As a result, it may infect and replicate in avian cells with higher efficiency than the mammalian ORFV. All vaccinations were performed by intramuscular injection to avoid overt shedding of the vaccines. As this infection route is rather unusual for NDV, it remains to be elucidated whether vaccination via mucosal routes is more efficient for these constructs [33].

Recently, Hameed et al. [11] described the immunization of cockatiels with recombinant PaBV-4 N protein without any viral vector component. Despite a detectable humoral immune response, the vaccination did not lead to reduced viral titres or protection against infection [11]. This is in line with our initial hypothesis that a potent cell-mediated immune response induced by infection-competent vaccines is required for protection against avian bornavirus infections [6,10] (Olbert et al., 2016; Runge et al., 2017). Surprisingly, in the study of Hameed et al. [11], the immunized birds hardly developed PDD-associated lesions and disease despite high viral loads in their tissues, while the majority of the unvaccinated controls succumbed to PDD. The authors speculate that their immunization triggered a mainly T_H_2-dominated response, which did not protect against subsequent challenge infection but prevented the induction of a pathogenic cell-mediated immune response. In line with this assumption, they demonstrated T-lymphocyte suppression by CsA treatment to prevent PDD in experimentally PaBV-2-infected cockatiels [11].

The confirmation that PDD is the result of an immunopathogenesis raised further questions regarding the possibility of triggering immunopathology by vaccination of persistently bornavirus-infected birds. Here we showed that experimentally PaBV-4 infected cockatiels immunized with MVA/PaBV-4 and NDV/PaBV-4 constructs did not develop exacerbated clinical disease and microscopic lesions, as compared to the control group receiving the parental vaccine strains. Although the health status of several birds worsened after immunization, particularly in the three weeks following the NDV application, these observations cannot be attributed to PaBV-4 vaccination, since they appeared in the groups receiving MVA/PaBV-4 and NDV/PaBV-4 or the parental vectors alike. Furthermore, clinical signs suggestive of PDD had been detectable in several birds already prior to immunization. Vaccination also did not result in reduced viral loads, indicating that the constructs are not applicable as a therapeutic vaccine. While MVA/PaBV-4 constructs provided a protective effect in naïve birds, they are apparently unable to trigger or modulate bornavirus-specific immunity in the face of an established infection, despite detectable induction of an immune response directed against the MVA vaccine backbone. This is in contrast to observations on BoDV-1 infection in experimental hosts, such as rats and mice, during which immunization of persistently infected animals induced immunopathogenesis [12].

As an interesting side aspect of this study, we could confirm previous observations of Runge at al. [10] that cockatiels show transient shedding of low amounts of viral RNA during the first three to five weeks after combined intramuscular and subcutaneous PaBV-2 inoculation. A similar phenomenon had been observed for experimental infection of common canaries (*Serinus canaria*) with canary bornavirus 1 and 2 [6,9,26]. These observations indicate that parenteral bornavirus inoculation may induce an early systemic phase of infection, followed by a temporal reduction or even elimination of the virus in peripheral organs and a subsequent resurgence. Intriguingly, Leal de Araujo et al. [34] did not observe early shedding or systemic distribution of viral RNA following subcutaneous PaBV-2 inoculation of cockatiels. It remains to be elucidated whether this discrepancy may be the result of a different PaBV-2 isolate, the different route of infection or a lower sensitivity of the employed detection assays. Interestingly, early shedding of viral RNA was not detected in cockatiels after experimental PaBV-4 infection in this or earlier studies [6,9].

In summary, our results show that MVA-based vaccines are effective candidates to protect parrots against avian bornavirus infections and associated diseases. Future work will have to determine the individual contribution of N, P or other bornavirus antigens to protection, the vaccine dose and the number of repetitive boosts required for a robust and long-lasting protection as well as the protective effect against more distantly related psittacine bornaviruses such as PaBV-5 or PaBV-6.

## Figures and Tables

**Table 1:** Infection parameters, clinical signs and macroscopic and microscopic lesions observed after PaBV-2 infection of vaccinated cockatiels (experiment 1).

Group	Anti-PaBV Titre at Challenge (Log iIFT units/mL)	PaBV-2 Shedding ^a^	Number of PaBV-2-pos. Organs ^b^	Clinical Disease ^c^ (Weeks p.c. ^d^)	Proven-tricular Dilatation	Mononuclear Infiltration Score						
Bird						Cere-Brum	Adrenal Gland	Heart	Crop	Proven-triculus	Gizzard	Duo-denum
NDV												
A1	4.1	+	11	-	-	+	n.a. ^e^	+++	+++	-	+++	-
A2	<2.6	-	4	-	-	-	+	+	-	-	+	-
A3	3.2	+	11	-	-	+	+++	+++	++	++	-	+++
A4	3.8	+	11	-	-	-	+++	+++	+	++	+++	++
MVA												
B1	4.7	-	1	-	-	-	-	-	-	-	-	-
B2	4.7	-	-	-	-	-	-	-	-	-	-	-
B3	3.8	-	-	-	-	-	n.a.	-	-	-	-	-
B4	4.1	-	-	-	-	-	-	-	-	+	-	-
ORFV												
C1	<2.6	+	11	-	-	-	+++	+++	+	-	-	-
C2	3.5	+	10	15–16	+	+++	n.a.	++	++	++	-	++
C3	3.8	(+) ^f^	3	-	-	-	-	-	-	-	-	-
C4	3.5	+	11	-	-	-	n.a.	-	-	-	-	+
Control												
D1	<2.6	+	11	-	-	-	-	-	++	+	-	-
D2	<2.6	-	8	-	-	-	n.a.	++	++	-	-	-
D3	<2.6	+	11	-	-	-	n.a.	++	+++	+++	+++	+++
D4	<2.6	-	3	-	-	-	n.a.	-	-	-	-	-

^a^ Detection of PaBV-2-specific RNA in cloacal swabs by RT-qPCR during week 8 to 16 after challenge infection. ^b^ Detection of PaBV-2-specific RNA by RT-qPCR. For each bird, 11 organs were analysed. ^c^ Clinical signs included slight apathy, ruffled feathers and weight loss. None of the birds were euthanized due to progressive disease. ^d^ p.c. = post challenge infection. ^e^ n.a. = not analysed. ^f^ transient shedding of low amounts of viral RNA.

**Table 2:** Clinical signs and microscopic lesions after immunization of persistently PaBV-4-infected cockatiels (experiment 2).

Group	Clinical Disease ^a^ (Weeks p.i. ^b^)		Death (Week p.i.)	Mononuclear Infiltration Score							
Bird	Before First Immunization	After First Immunization ^c^		Cere-brum	Cere-bellum	Adrenal Gland	Heart	Pan-creas	Crop	Proven-triculus	Gizzard
Immunized											
#1	-	-	-	++	+	+++	++	++	-	-	-
#2	-	-	-	-	-	n.a. ^d^	++	++	-	-	-
#4	-	-	-	-	-	-	++	++	++	+++	+
#5	6–8	16–18	18	+	+	++	++	n.a.	+++	++	++
#6	5	17–18	-	-	-	n.a.	++	-	+	+	+
#12	9	-	-	-	-	+++	++	+	++	+	-
Control											
#3	5	-	-	+++	+++	+++	++	+++	+++	+++	+++
#7	5–8, 12	13–22	-	-	-	++	+++	+++	++	++	+
#8	-	-	-	-	-	n.a.	++	+	+	+++	-
#9	-	-	-	+	-	+	+	+++	+	++	+
#10	-	18	-	+	-	++	+++	++	+++	+++	+
#11	-	-	-	-	-	-	-	-	-	+	+

^a^ Clinical signs included slight apathy, ruffled feathers and weight loss. ^b^ p.i. = post inoculation with PaBV-4. ^c^ Birds of the immunized group were immunized in weeks 12 and 18 p.i. with a mixture of rMVA/PaBV-4/N and P and in week 15 p.i. with rNDV PaBV-4/N vaccines. At the same time points, the control group received the respective parental vaccine strain. ^d^ n.a. = not analysed.

## Supplementary Materials

The following are available online at https://www.mdpi.com/1999-4915/11/12/1130/s1, Figure S1: Schematic illustration of a transfer plasmid used to generate ORFV vector vaccines; Figure S2: Recombinant ORFV vector vaccines express PaBV-4 N and P antigens in infected Vero cells; Figure S3: Infection and expression of PaBV-4 antigens by ORFV constructs in avian cells; Figure S4. Shedding of NDV, MVA and ORFV vaccine viruses in cockatiels after vaccination (experiment 1); Figure S5. Proventricular dilatation in a vaccinated cockatiel following PaBV-2 challenge infection (experiment 1); Figure S6. Grouping of persistently PaBV-4-infected cockatiels (experiment 2); Figure S7. Clinical scores and gross lesions in PaBV-4-infected and subsequently immunized birds (experiment 2); Figure S8. Development of NDV- and MVA-reactive antibodies in persistently PaBV-4-infected cockatiels (experiment 2).
