# Supplementary material for: Recombinant Modified Vaccinia Virus Ankara (MVA) Vaccines Efficiently Protect Cockatiels Against Parrot Bornavirus Infection and Proventricular Dilatation Disease
Source: Viruses. 2019 Dec 6;11(12):1130. doi: 10.3390/v11121130 (PMC6950018; doi:10.3390/v11121130)
Supplement: Supplementary file 1 [file viruses-11-01130-s001.pdf]

viruses

MDPI

Supplementary material

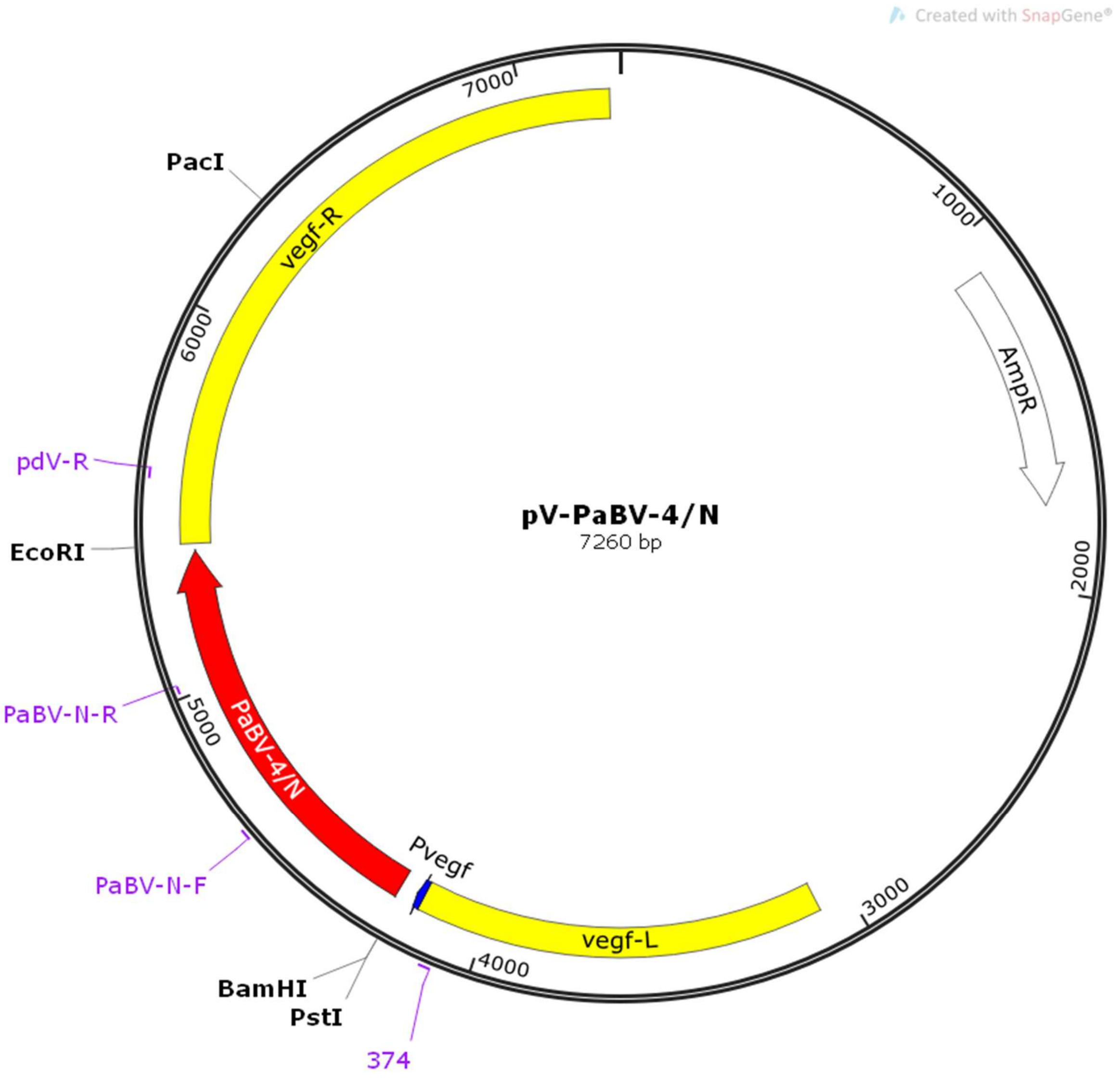

Figure S1. Schematic illustration of a transfer plasmid used to generate ORFV vector vaccines. The open reading frames of the nucleoprotein (N) and phosphoprotein (P) genes of parrot bornavirus (PaBV-4) were cloned as BamHI-EcoRI DNA fragments into the transfer plasmid pdVRec, which allows foreign gene insertion into the vegf-e gene of ORFV vector D1701-V-CD4-D12-mCherry as described [1, 2] to generate two transfer plasmids (pV-PaBV-4/N, pV-PaBV-4/P). Pvegf (blue): vascular endothelial growth factor (VEGF) promotor. vegf-R, vegf-L (yellow): right and left homologous VEGF regions. AmpR (white): ampicillin resistance gene. Selected restriction sites and primer annealing sequences are indicated. The figure was constructed using SnapGene (GSL Biotech; available on snapgene.com).

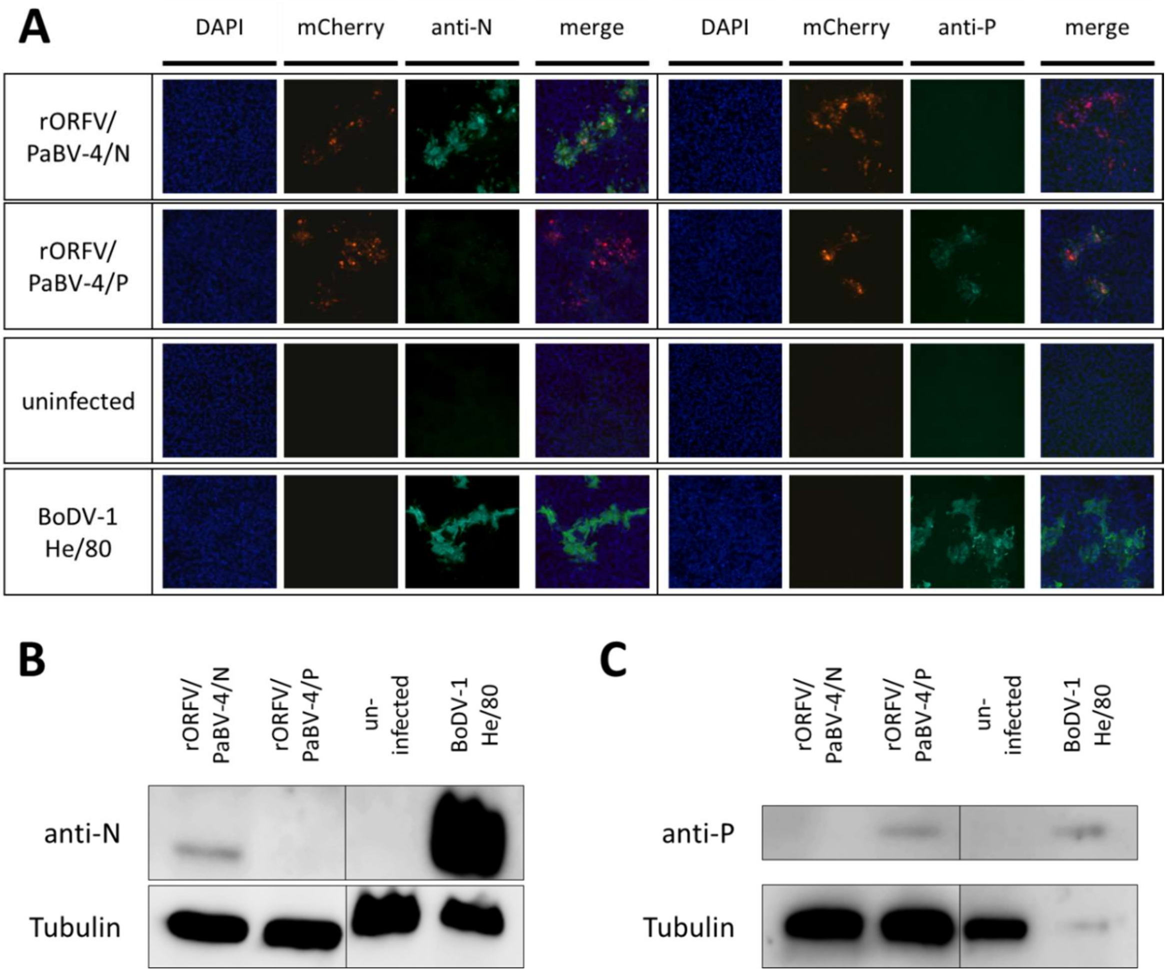

Figure S2. Recombinant ORFV vector vaccines express PaBV-4 N and P antigens in infected Vero cells. Vero cells were infected with rORFV/PaBV-4/N and rORFV/PaBV-4/P (multiplicity of infection [MOI] of 0.5). Uninfected Vero cells and cells persistently infected with BoDV-1 strain He/80 were included as controls. (**A**) Immunofluorescence staining was performed at 72 hours post infection with polyclonal rabbit sera directed against PaBV-2 N (left panel) or BoDV-1 P (right panel) followed by incubation with polyclonal goat-anti-rabbit serum conjugated with Cy2 (green). ORFV-infected cells are marked by the fluorescent marker mCherry (red). DAPI staining (blue) indicates cell nuclei. (**B**, **C**) Cell lysates were harvested for Western blotting at 48 hours post infection. Expression of bornavirus proteins was detected using polyclonal rabbit sera directed against PaBV-2 N (**B**) or BoDV-1 P (**C**). Rabbit-anti-tubulin staining was used as the loading control.

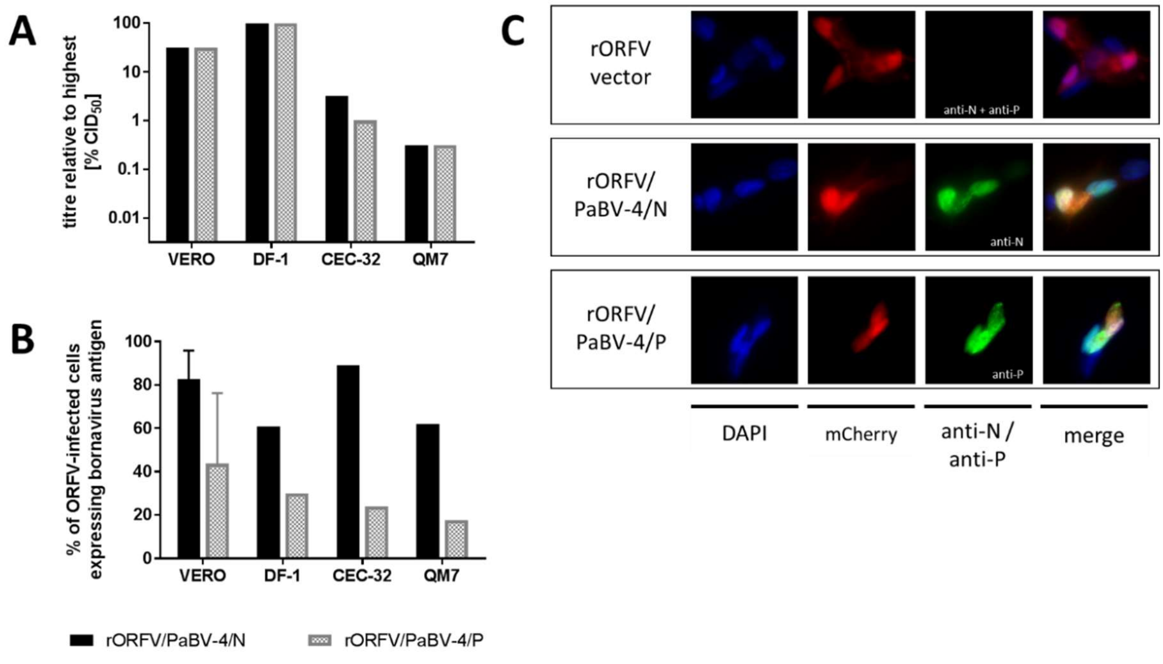

Figure S3. Infection and expression of PaBV-4 antigens by ORFV constructs in avian cells. (**A**) Avian and mammalian cell lines were infected with dilution series of constructs rORFV/PaBV-4/N and rORFV/PaBV-4P. Median culture infectious doses (CID_{50}) were calculated and are presented relative to the highest titer obtained for each construct. (**B**, **C**) Avian and mammalian cell lines were infected with rORFV/PaBV-4/N and rORFV/PaBV-4P (Vero: MOI = 1; avian cells: MOI = 5). After incubation for 24 hours (**B**) or 40 hours (**C**), immunostaining was performed with antibodies directed against PaBV-2 N or BoDV-1 P, respectively. (**B**) Bornavirus antigen-positive cells were quantified by flowcytometry and presented as percentage of ORFV-infected cells (mCherry-positive). (**C**) Bornavirus antigen expression in avian QM7 cells. The parental vector strain (D1701-V-CD4-D12-mCherry) not carrying bornavirus genes served as a control. Vero: African green monkey kidney cells; DF-1: chicken fibroblasts; CEC-32: quail fibroblasts; QM7: quail smooth muscle cells.

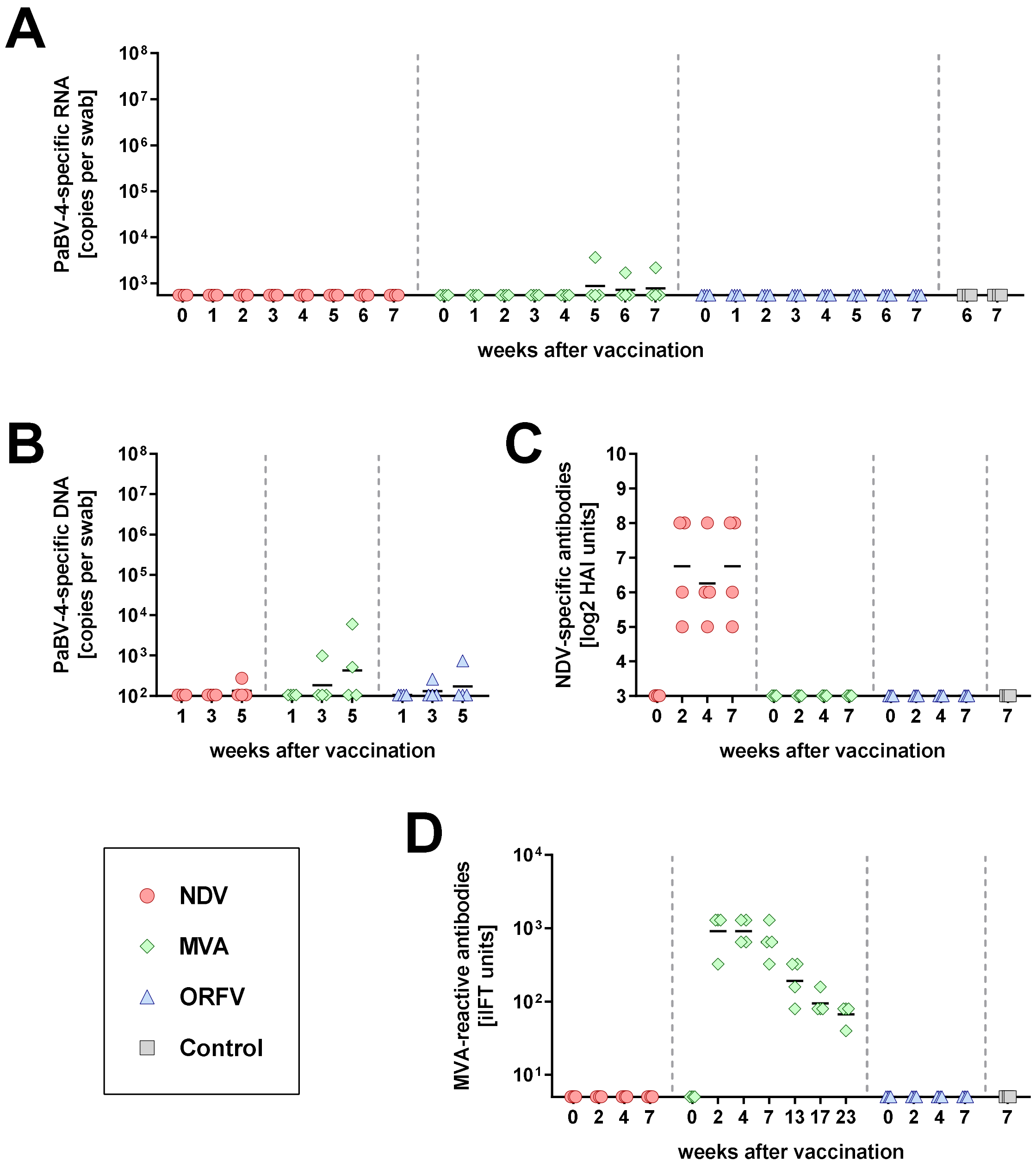

Figure S4. Shedding of NDV, MVA and ORFV vaccine viruses in cockatiels after vaccination (experiment 1). Groups of four cockatiels were vaccinated three times with mixtures of rNDV/PaBV-4/N & P (NDV), rMVA/PaBV-4/N & P (MVA) or rORFV/PaBV-4/N & P (ORFV), respectively. A fourth group (Control) was not vaccinated. (**A**, **B**) Cloacal swabs were collected in weekly intervals and PaBV-4 P-specific RNA (**A**) or DNA (**B**) was quantified by RT-qPCR. (**C**, **D**) Serum samples were collected and tested for the presence of NDV-specific antibodies by hemagglutination inhibition (HAI) test (**C**) or MVA-reactive antibodies by indirect immunofluorescence test (iIFT; **D**). Dots represent results of individual birds. Horizontal lines indicate geometric means of each group at the respective time points. The position of the X axis indicates the detection limit of the respective test.

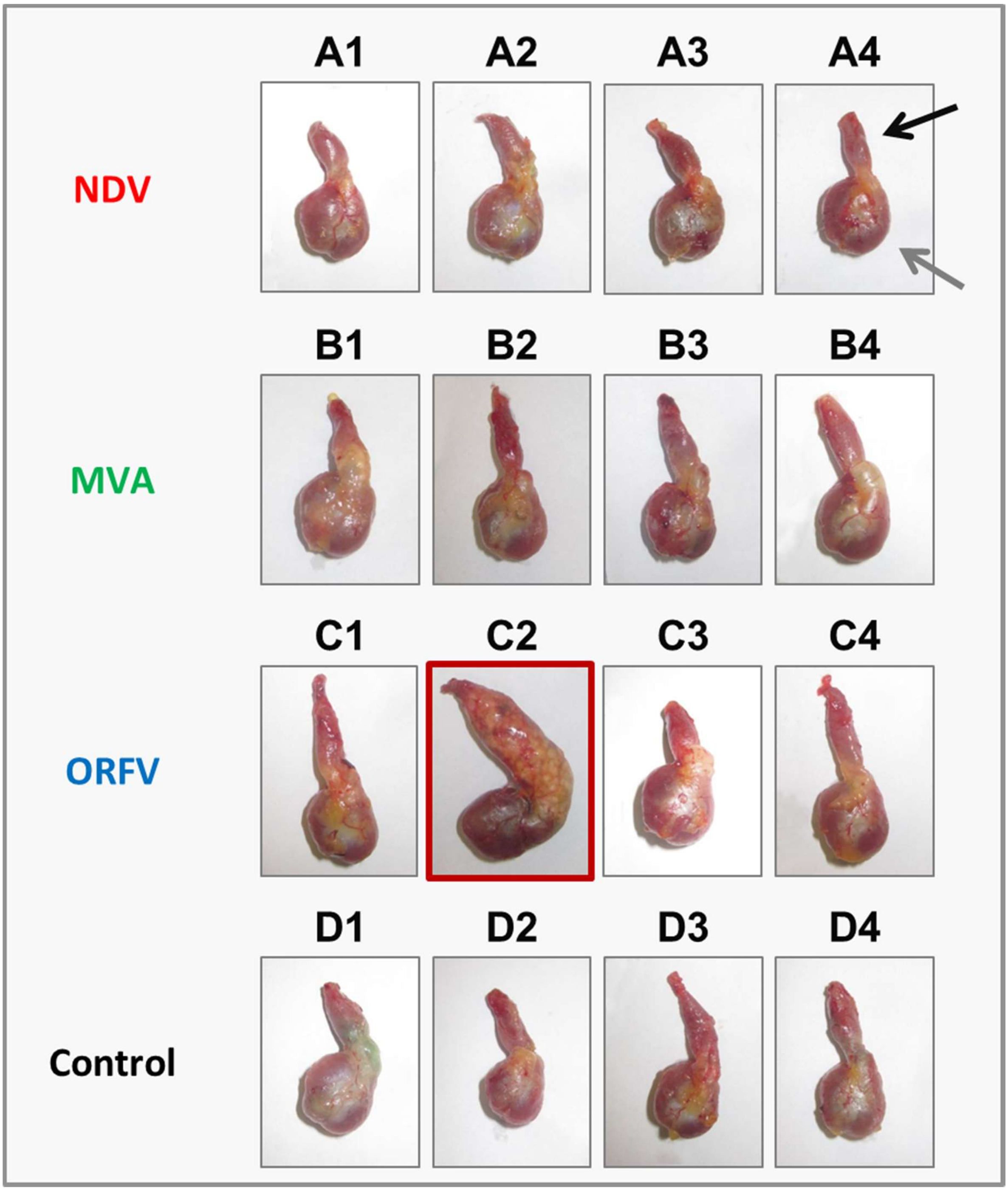

Figure S5. Proventricular dilatation in a vaccinated cockatiel following PaBV-2 challenge infection (experiment 1). Three groups (NDV, MVA, ORFV) of four cockatiels each were immunized three times with the indicated vector vaccines expressing PaBV-4 N and P genes. A fourth group (Control) was not vaccinated. Three weeks after the third vaccination, all groups received a heterologous PaBV-2 #17684 challenge infection. All birds were euthanized and necropsied at 16 weeks after challenge infection. Bird C2 (red box) exhibited a marked dilatation of the proventriculus, typical of proventricular dilatation disease (PDD), while all other birds did not express apparent macroscopic lesions. Arrows indicate proventriculus (black) and gizzard (grey) of a representative bird.

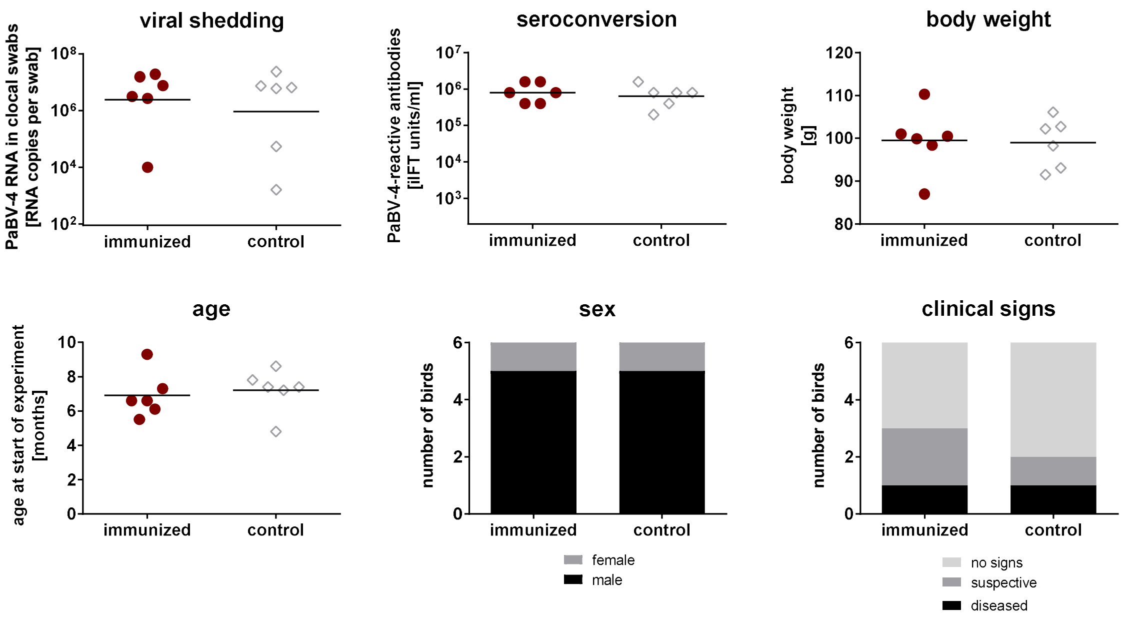

Figure S6. Grouping of persistently PaBV-4-infected cockatiels (experiment 2). Twelve cockatiels were experimentally inoculated with PaBV-4 #6758. At week 12 after infection (p.i.), the birds were divided into two equal groups based on the following parameters: shedding of viral RNA in week 12 p.i., PaBV-4-reactive antibodies in week 12 p.i., body weight in week 12 p.i., age, sex and clinical signs observed since the beginning of the experiment.

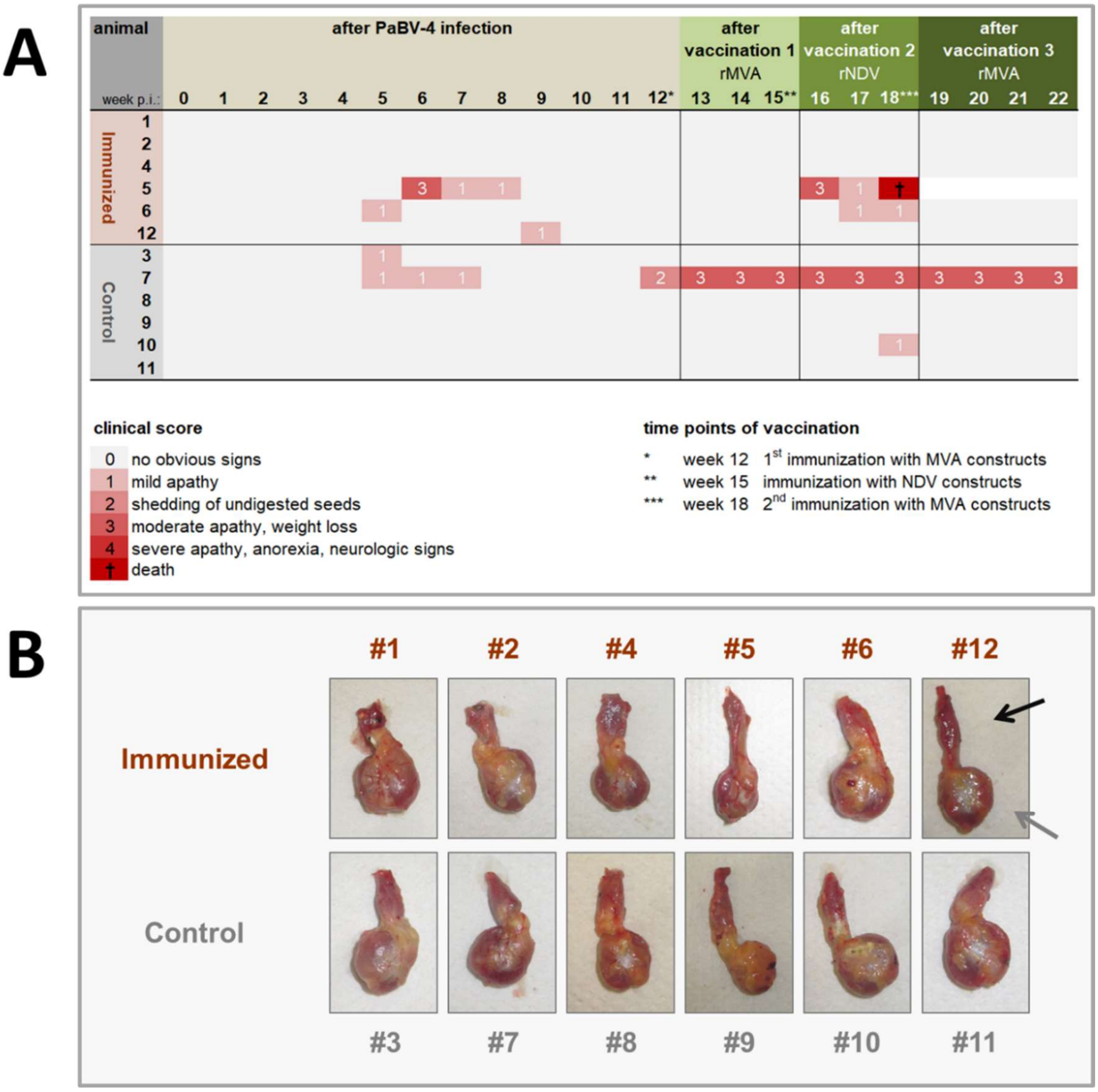

Figure S7. Clinical scores and gross lesions in PaBV-4-infected and subsequently immunized birds (experiment 2). Two groups of six cockatiels each were experimentally inoculated with PaBV-4 #6758. Subsequently, one group (Immunized) was immunized twice with mixtures of rMVA/PaBV-4/N and P at 12 and 18 weeks after infection and with rNDV/PaBV-4/N and P at 15 weeks after infection. The second group (Control) received the respective parenteral vaccine strains not expressing bornavirus antigens. (**A**) All birds were monitored daily and clinical signs were recorded as clinical scores. Bird #5 succumbed to disease during week 18 after PaBV-4 infection. (**B**) All remaining birds were euthanized and necropsied at 22 weeks after infection. None of the birds exhibited apparent macroscopic lesions. Arrows indicate proventriculus (black) and gizzard (grey) of a representative bird.

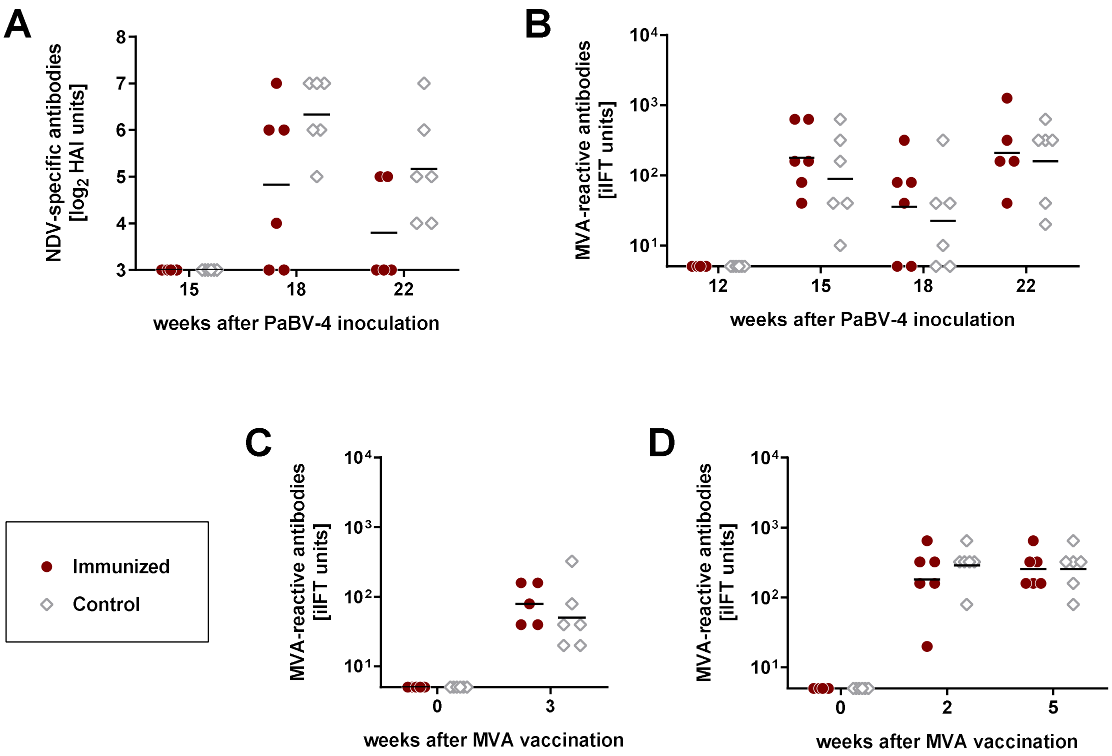

Figure S8. Development of NDV- and MVA-reactive antibodies in persistently PaBV-4-infected cockatiels (experiment 2). (**A**, **B**) Two groups of six cockatiels each were experimentally inoculated with PaBV-4 #6758. Subsequently, one group (Immunized) was immunized twice with mixtures of rMVA/PaBV-4/N and P at 12 and 18 weeks after infection and with rNDV/PaBV-4/N and P at 15 weeks after infection. The second group (Control) received the respective parenteral vaccine strains not expressing bornavirus antigens. Serum samples were collected and tested for the presence of antibodies directed against the vaccine vectors NDV and MVA. (**A**) NDV-specific hemagglutination inhibiting (HAI) antibodies. (**B**) MVA-reactive antibodies determined by iIFT. (**C**, **D**) MVA-reactive antibodies developed by cockatiels vaccinated with rMVA/PaBV-4/N & P and rNDV/PaBV-4/N & P (Immunized) or with the respective parenteral strains (Control) in previously published experiments [3, 4] are shown for comparison. (**C**) Cockatiels of both groups received a single injection of MVA constructs and antibody response was determined three weeks later [3]. (**D**) In a further experiment, all birds received two MVA vaccinations at an interval of two weeks. Serum titers were determined at two weeks after the first and three weeks after the second injection [3, 4]. For detailed description of the experimental design and titers of NDV-reactive HAI antibodies of these birds, refer to Olbert et al. [3]. Dots represent results of individual birds. Horizontal lines indicate geometric means of each group at the respective time points. The position of the X axis indicates the detection limit of the respective test.

References:

1. Fischer, T.; Planz, O.; Stitz, L.; Rziha, H. J., Novel recombinant parapoxvirus vectors induce protective humoral and cellular immunity against lethal herpesvirus challenge infection in mice. *Journal of virology* **2003,** 77, (17), 9312-23. doi: 10.1128/jvi.77.17.9312-9323.2003
2. Rziha, H. J.; Rohde, J.; Amann, R., Generation and Selection of Orf Virus (ORFV) Recombinants. *Methods Mol Biol* **2016,** 1349, 177-200. doi: 10.1007/978-1-4939-3008-1_12
3. Olbert, M.; Römer-Oberdörfer, A.; Herden, C.; Malberg, S.; Runge, S.; Staeheli, P.; Rubbenstroth, D., Viral vector vaccines expressing nucleoprotein and phosphoprotein genes of avian bornaviruses ameliorate homologous challenge infections in cockatiels and common canaries. *Sci Rep* **2016,** 6, 36840. doi: 10.1038/srep36840
4. Runge, S.; Olbert, M.; Herden, C.; Malberg, S.; Römer-Oberdörfer, A.; Staeheli, P.; Rubbenstroth, D., Viral vector vaccines protect cockatiels from inflammatory lesions after heterologous parrot bornavirus 2 challenge infection. *Vaccine* **2017,** 35, (4), 557-563. doi: 10.1016/j.vaccine.2016.12.022
